# A Transient Expression of *Prospero* Promotes Cell Cycle Exit of *Drosophila* Postembryonic Neurons through the Regulation of *Dacapo*

**DOI:** 10.1371/journal.pone.0019342

**Published:** 2011-04-28

**Authors:** Jordi Colonques, Julian Ceron, Heinrich Reichert, Francisco J. Tejedor

**Affiliations:** 1 Instituto de Neurociencias, CSIC-UMH, Alicante, Spain; 2 Biozentrum, University of Basel, Basel, Switzerland; National Institutes of Health (NIH), United States of America

## Abstract

Cell proliferation, specification and terminal differentiation must be precisely coordinated during brain development to ensure the correct production of different neuronal populations. Most *Drosophila* neuroblasts (NBs) divide asymmetrically to generate a new NB and an intermediate progenitor called ganglion mother cell (GMC) which divides only once to generate two postmitotic cells called ganglion cells (GCs) that subsequently differentiate into neurons. During the asymmetric division of NBs, the homeodomain transcription factor PROSPERO is segregated into the GMC where it plays a key role as cell fate determinant. Previous work on embryonic neurogenesis has shown that PROSPERO is not expressed in postmitotic neuronal progeny. Thus, PROSPERO is thought to function in the GMC by repressing genes required for cell-cycle progression and activating genes involved in terminal differentiation. Here we focus on postembryonic neurogenesis and show that the expression of PROSPERO is transiently upregulated in the newly born neuronal progeny generated by most of the larval NBs of the OL and CB. Moreover, we provide evidence that this expression of PROSPERO in GCs inhibits their cell cycle progression by activating the expression of the cyclin-dependent kinase inhibitor (CKI) DACAPO. These findings imply that PROSPERO, in addition to its known role as cell fate determinant in GMCs, provides a transient signal to ensure a precise timing for cell cycle exit of prospective neurons, and hence may link the mechanisms that regulate neurogenesis and those that control cell cycle progression in postembryonic brain development.

## Introduction

In order to give rise to the diversity and specificity of cells types in the brain, cell proliferation, specification and terminal differentiation must be precisely coupled in space and time during development to ensure the correct number of cells in different populations and specify their resulting connectivity. Recent work has shown that the postembryonic central nervous system (CNS) of *Drosophila* is a suitable experimental model to study the genetic basis of some of these processes, including neural proliferation, cell lineage specification, and asymmetric division of neural progenitor cells, as well as tumourigenesis if these processes are perturbed [reviewed by 1]–[3].

**Figure 1:**
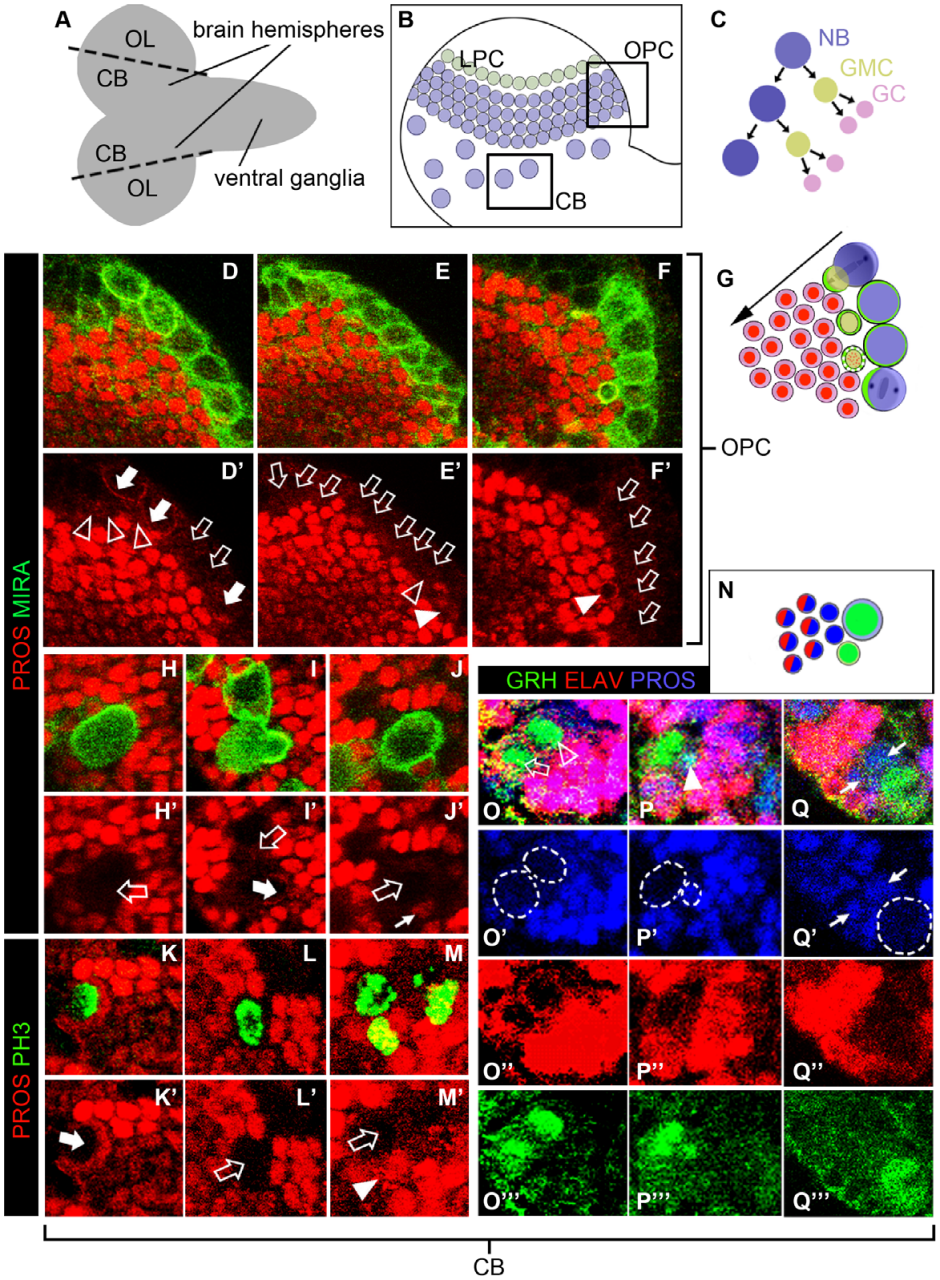
Cellular Pattern of PROSPERO Protein Expression in the larval brain. A–C. Morphology, cellular organization and pattern of division in the larval CNS. A. Schematic representation of a late larval CNS which is composed of two brain hemispheres and ventral ganglia. The central brain (CB) develops in the medial regions of each hemisphere, while the optic lobes (OL) primordia are located laterally. B. Schematic drawing of a brain hemisphere showing the scattered distribution of CB NBs in the medial part and the LPC (Lamina precursor cells) and OPC (outer proliferation centre) neuroepithelia located laterally. C. The typical pattern of division of type I CB and OPC NBs. Each NB divides asymmetrically several times to generate a new NB and a ganglion mother cell (GMC) that divides once to generate two postmitotic ganglion cells (GCs). D–F. Co-expression and subcellular co-localizacion of PROS and MIRA in the OPC. Late third instar larval brains were immunostained with PROS and MIR antisera and analyzed by confocal microscopy. Images focusing on the same region of the OPC in three confocal sections of the same OL taken at different ventro-dorsal levels. Note the strong PROS labeling in the nuclei of the GCs located below the superficial MIR+ NBs which, apart from a few exceptions (filled arrows), mostly lack PROS (empty arrows). Similarly, in only a few GMCs (medium size MIR+ cells located in the first internal layer, just below the NBs) did MIR and PROS co-localize (filled arrowheads) while in most of them PROS was hardly detected (empty arrowheads). G. Representation of the OPC showing the NBs located on the surface, which generate their progeny radially inside de OL. These NBs localize asymmetrically MIRA during their division. The progeny express nuclear PROS. H–J. Representative examples of CB NBs and their nearby progeny immunostained with PROS and MIRA. H. Most frequently NBs with asymmetric MIRA lacks PROS immunostaining (empty arrow). I. Nevertheless, in some cases some NBs with asymmetric MIRA showed PROS signal (filled arrow) but this is much weaker than that observed in the nuclei of the surrounding GCs. J. Notice that PROS signal is also weaker in the nuclei of single weak MIRA+ cells attached to the NBs (small arrows, putative GMCs) than in the nuclei of the surrounding GCs. K–M. Representative examples of mitotic CB NBs and their nearby progeny immunostained with PROS and PH3. Only a few among the mitotic CB NBs showed asymmetric PROS signal (K, filled arrow) while most mitotic NBs (L, M; empty arrows. showed low (if any) PROS immunosignal. Some mitotic GMCs exhibited nuclear PROS signal (M; arrowhead) although weaker than the surrounding GCs. N, Representation of a CB NB lineage which summarizes the pattern of expression of GRH, PROS and ELAV showed in panels O–P. O–P, Representative examples of CB NBs and their nearby progeny immunostained with GRH, ELAV and PROS. O. One GRH+ NB (empty arrow) and its neighbor GRH+ GMC (empty arrowhead) lack PROS labeling while all the surrounding ELAV+ GCs exhibit strong PROS signal. P. One of the less frequent cases in which a GRH+ GMC (filled arrowhead) located close to a NB, exhibited PROS signal. Q. Image showing a pair of GRH-/PROS+/ELAV- cells (small arrows) attached to a NB. Note that the rest of the progeny is GRH-/PROS+/ELAV+.

The CNS of *Drosophila* is composed of two brain hemispheres and the ventral ganglia. The adult CB develops in the medial regions of each hemisphere, while the adult OLs develop laterally (see Fig. 1A, B for a schematic summary).

Most of the cells comprising the adult brain are generated from progenitor cells called neuroblasts (NBs) that become quiescent at the end of embryonic development and that re-enter the cell cycle at different times during larval development depending on the region and cell type.

Proliferation during postembryonic development of the OL and CB has been studied extensively. Each optic lobe (OL) is generated from three neuroepithelia called the LPC (Lamina precursor cells), OPC (outer proliferation centre) and IPC (inner proliferation centre) [4], [5] which give rise to the adult lamina, medulla, and lobula, respectively. OPC and IPC neuroepithelial progenitors switch from symmetric, proliferative to asymmetric, neurogenic divisions during the third instar stage [1], [6], [7]. Thus, most neurogenesis takes place in the OL at the end of larval development [4], [5], [8], [9].

By contrast, most of cells of the adult CB originate from a number of scattered NBs located medially in the hemispheres, which proliferate from the first instar stage until the beginning of pupal development [4], [8], [10]–[13]. Two main different types of NBs have been found in the CB. Most of the NBs (Type I) follow patterns of proliferation similar to those of embryonic NBs, although they produce more cells in each lineage. Thus, each Type I NB divides asymmetrically several times to generate in each division a new NB and an intermediate GMC progenitor which divides once to generate two postmitotic daughters called ganglion cells (GCs) that differentiate into neurons [3], [6], [14] (Fig. 1C). A smaller group of Type II NBs has a different proliferative mode that involves intermediate progenitors with transit amplifying cell divisions [15]–[17].

During each division of embryonic NBs, the homeodomain transcription factor, PROSPERO (PROS) [18], due to its binding to the carrier protein MIRANDA (MIRA), is asymmetrically segregated from the parent NBs to its daughter GMC where it plays a key role as cell fate determinant (reviewed in [19]). In the GMC, PROS translocates to the nucleus and acts to repress the expression of cell-cycle regulators [20] and activate genes that direct terminal differentiation of neurons [21]. Recent work indicates that expression and action of PROS is similar in postembryonic Type I NBs and their GMC daughter cells [1], [15], [17], [22]–[24]. However, there are several differences in the cellular pattern of PROS expression between embryonic and larval NB lineages [6] as well as in the phenotype of *pros* mutants in the embryonic and larval CNS [21]–[26]. Given these differences in expression and phenotypes, it seems likely that there might also be differences in the functional roles played by PROS during embryonic versus postembryonic CNS development.

To investigate this, we have performed a genetic, cellular and molecular analysis of the roles played by PROS in neural proliferation and neurogenesis during postembryonic development of the *Drosophila* brain. We focused on CB type I and OPC NB lineages. We find that a marked transient upregulation of PROS expression occurs in postmitotic GCs shortly after the division of their parent GMC. We provide evidence for the fact that this transient PROS upregulation inhibits cell cycle progression in the GCs. Furthermore, we identify the pan-neural bHLH transcription factor DEADPAN (DPN) and the cyclin kinase inhibitor DACAPO (DAP), as candidate downstream effectors of PROS in this function. In view of these findings, we discuss the implications of different roles of PROS in embryonic versus post-embryonic neurogenesis of *Drosophila*.

## Materials and Methods

### *Drosophila* strains and mosaic analysis

All the fly stocks used in this study were derived from *Drosophila melanogaster* and they were raised at 25°C on standard medium, except when particular temperature conditions were required (see below). The *wt* strains used were *Berlin* and *Canton-S*. Fly stocks carrying mutations, transgenes and recombinant chromosomes were: *pros^voila24^/TM6,Sb* [27], *UAS-pros* and *pros^v17^* (Doe et al., 1991), *UASmCD8::GFP*, *UAS-nlslacZ*, *hs-Gal4/TM3* (Bloomington Stock Center), *c820-Gal4* and *c831-Gal4* [28].

To induce the overexpression of PROS, *hs-Gal4;UAS-pros* larvae were grown at 17°C until mid third instar stage. The level of PROS expression remained apparently normal under these conditions. Then, a short heat shock was applied at 37°C and the larvae were grown at 29°C for 15 h until wandering larval stage. Increased expression of PROS begins 6 h after the heat shock. Similarly, *c831Gal4;UAS-pros* larvae were grown at 17°C until mid third instar stage. Then, the temperature was shifted to 29°C for 12–15 h until wondering larval stage.

Clonal mosaic analysis was carried out by generating mitotic *wt* or *pros^v17^* clones by the MARCM technique [29]., crossing *hsFLP*; *tubP-GAL4/Cy(Actin-GFP)*; *FRT82B,tubP-GAL80/(Tm6,Tb)* flies with either *+/+*; *UAS-nlacZ*, *UAS-CD8::GFP/(Cy)*; *FRT82B/(TM6,Tb) or +/+*; *UAS-nlacZ, UAS-CD8::GFP/(Cy(Actin-GFP))*; *FRT82B*, *pros^v17^/(TM6,Tb)*, respectively [30]. The production of *wt* and *pros^v17^* clones was induced by giving a heat shock of 1 h at 37°C to third instar larvae 24 h prior to dissection at wandering larval stage.

### Bromodeoxyuridine (BrdU) labeling

*In vitro* BrdU labeling of whole-mount larval brains was carried out essentially as described previously [6] but with incubation times of 5–10 min. Larval brains of late third instar (wandering) larvae) were dissected in Ringer's solution and fixed for 3 min with modified Carnoy's fixative followed by 75% EtOH for 30 min. After rehydration, the samples were denatured by treatment with 2 N HCl for 40 min and they were then neutralized by washing with phosphate buffer saline (PBS) before proceeding to the immunocytochemical analysis with an anti-BrdU antiserum (Beckton-Dickinson) and a horse radish peroxydase (HRP) coupled secondary antibody visualized with diaminobenzidine (DAB).

### Immunohistochemistry

Larval brains were dissected out in PBS and fixed for 30 min on ice with 4% paraformaldehyde in PBS, and then for a further 30 min with 4% paraformaldehyde, 0.1% Triton X-100 in PBS. After washing in PBS, the larval brains were sometimes dehydrated with 100% methanol and rehydrated stepwise to PBS. Brains were incubated with antisera overnight at 4–8°C in PBS containing 5% normal goat serum, 0.1% Triton X-100 and 0.02% Sodium Azide. The following primary antibodies were used: rabbit anti-Beta-Gal (Sigma immunochemicals), anti-DE-CADHERIN (a kind gift from T. Uemura); anti-activated CASPASE3 (Cell Signaling Technology), mouse and rat anti-CYCLIN E (a kind gift H. Richardson), anti-DAP (a kind gift from I.K. Hariharan ), guinea pig anti-DPN (a kind gift from J. Skeath), rat anti-ELAV (Developmental Studies Hybridoma Bank), mouse and rabbit anti-GFP (Invitrogen), rabbit anti-GRH (Almeida and Bray, 2005), rabbit anti-MIR (a kind gift from C. Gonzalez); rabbit anti-Phosphohistone-3 (PH3) (Upstate Biotechnology); mouse anti-PROS (MR1A, a kind gift from C. Doe). Fluorescent-labeled secondary antibodies (Jackson Immnunochemicals) were used according to the manufacturer's recommendations. For the detection of PROS, we used biotinylated secondary antibodies and Cy2 or Cy3 conjugated streptavidin or HRP-coupled secondary antibodies followed by Tyramide detection (TSA, Perkin Elmer). Immunolabeled samples were analyzed on a Leica TCS-SL spectral confocal microscope.

### Fluorescent *in situ* hybridization (FISH)

Digoxigenin (DIG) or Fluorecein DNA labeled probes were synthesized by PCR. To prepare a *pros* probe, we used a 267 bp fragment corresponding to positions 4036–4303 of *pros* cDNA [26]. For *dap* we used a 253 bp fragment corresponding to positions 911–1163 of *dap* cDNA [31]. The hybridized probes were detected with HRP-coupled anti-DIG (Roche) and rabbit anti-Fluorescein (Molecular Probes) antibodies and visualised with the TSA-Cy2 or Cy3 detection kits (Perkin Elmer). For double FISH, after hybridization with the DIG and Fluorescein labeled probes, binding of the HRP-coupled anti-DIG and anti-Fluorecein antibodies, the corresponding fluorescent TSA reactions were performed sequentially, inactivating the HRP activity with H_2_O_2_ in between the detection of the two antibodies.

### Analysis of cell death

Apoptotic cell death was monitored in the late third instar larval brains by immunohistochemical analysis of activated CASPASE-3, as described above.

## Results

### *prospero* expression is upregulated in new born post-embryonic neurons

Previous work on neurogenesis in the embryonic CNS has shown that PROS protein is expressed in NBs and GMCs but is lacking in postmitotic neural GCs [25], [26]. In contrast, in postembryonic neurogenesis, high levels of PROS protein expression have been found in postmitotic GCs [6]. This suggests that PROS might have a novel function in postembryonic GCs. To investigate this, we first carried out a detailed immunocytochemical study of PROS expression in OPC and CB NBs in the third instar larval brain. For this we combined PROS immunolabeling with the mitotic PH3, the two NB/GMC markers, MIRANDA(MIRA) and GRAINYHEAD (GRH) [32], and the pan-neuronal marker ELAV [33]. Together these markers made it possible to distinguish between PROS expression in NBs, GMCs and GCs (prospective neurons) (see Table 1). We limited our study to the OPC and CB Type I NB lineages since Type II [15], [17] and mushroom bodies [34] NBs do not express PROS.

**Table 1:** Differential expression of molecular markers in NBs, GMCs, and GCs.

	MIR	PH3	GRH	PROS	ELAV
NB	++	−	Nuclear+++	−	Cortex+/−
MitoticNB	Asymmetric+++	+++	Nuclear++	Asymmetric+/−	Cortex+/−
GMC	Cortex+++	−	Nuclear++	Cortex+/−	Cortex+/−
MitoticGMC	Cortex++	+++	n.d.	Nuclear+/−	Cortex+/−
New bornGCs	Cortex+/−	−	−	++	−
GCs		−	−	+++	Nuclear+++

*The level of immunostaining of the molecular markers in the different cell identities is described in an arbitrary comparative scale: absent (−), weak (+), intense (++), very intense (+++).*

In the cells of the OPC, a marked difference in the level of PROS expression was observed in that GCs showed much stronger PROS expression than NBs and GMCs (Fig. 1D–F). Thus, high levels of PROS immunolabeling were consistently seen in the nuclei of GCs located inside the OPC. In contrast, relatively low levels of cortically localized and asymmetric PROS (together with MIRA) immunolabeling were observed in a subset of the NBs while no detectable PROS immunolabeling was observed in the remaining NBs. Similarly, the majority of the GMCs in the OPC had relatively low (or even undetectable) PROS immunostaining. Comparable findings were obtained for the CB (Fig. 1H–Q). Thus, strong and consistent PROS immunolabeling was detected in the nuclei of the postmitotic (ELAV-positive) GC progeny (Fig. 1O–Q). In contrast, relatively low (or undetectable) levels of PROS immunolabeling were seen in the majority of NBs, even in those that have asymmetrically localized MIRA (Fig. 1H–J) or expressed the mitotic marker PH3 (Fig. 1K–M). Relatively low (or undetectable) levels were also seen in most GMCs, independent of their mitotic status. Interestingly, we also frequently observed a pair of PROS+/GRH-/ELAV- cells attached to a NB (Fig. 1Q). According to this molecular marker profile, we identified these cells as recently born GCs.

Taken together, these findings indicate that in the larval brain the level of PROS expression is substantially higher in postmitotic GCs as compared to their NB and GMC progenitors. This markedly higher expression level in the progeny is unlikely to be due to the persistence of the little PROS protein detected in the GMC after being divided into the two daughter GCs. We therefore hypothesized that *pros* expression might be upregulated in new born neurons of the OPC and CB.

**Figure 2:**
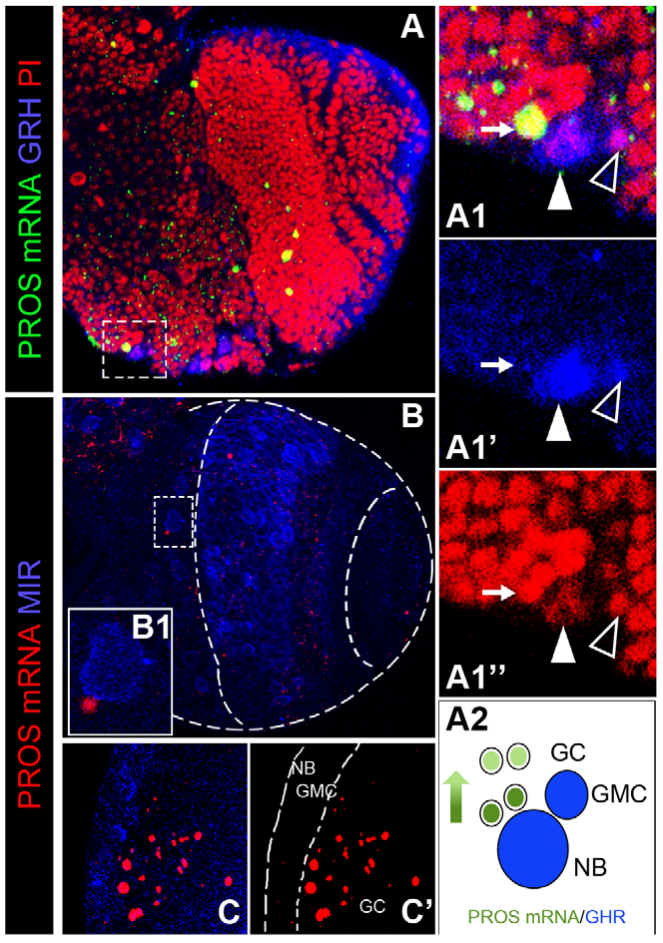
Expression of prospero mRNA in the larval brain. A. Confocal projection taken through a late larval brain hemisphere from a ventro-anterior orientation showing PROS mRNA expression in numerous scattered cells in the OPC and CB, GRH expression, and counterstaining with Propidium Iodide (PI). A1. High magnification view of the cell cluster framed in A showing a single PROS mRNA expressing cell (small arrow) located in close vicinity to a large NB (arrowhead) and a GMC (empty arrowhead), both expressing GRH. A2. Schematic representation of the cellular expression pattern of *pros*. B. Similar confocal projection as in A showing PROS mRNA and MIR expression. B1. High magnification of the CB cell cluster framed in B showing a single PROS mRNA expressing cell located near a large MIR+ NB. C. Confocal projection over 30 µm taken from a frontal orientation of the OPC. Notice the presence of several PROS mRNA expressing cells located underneath the layer of MIR+ cells.

To test this notion, FISH was used to monitor the expression of *pros* mRNA together with the above mentioned molecular markers to distinguish cellular identities. This FISH analysis revealed a punctate distribution of *pros* mRNA in numerous GC-like cells in the OPC and CB (Fig. 2). Thus, in the CB PROS mRNA signal was most often observed in small MIRA-/GRH- cells located in the vicinity of NBs (Fig. 2A, B). In the OPC, we found labeled cells located in the GC layers immediately beneath the external layer of (MIRA+) NBs and GMCs but rarely in more deep layers of older (differentiating) neurons, and we did not find consistent signal in most larval OL NBs (Fig. 2C). Nevertheless, we can not rule out the presence of *pros* transcripts in these NBs below our detection threshold. Together, these results support the idea that *pros* mRNA transcription is transiently upregulated in recently generated neurons (GCs). In further support of the transient nature of PROS expression in GCs, we observed that PROS protein signal in the OPC is markedly weaker after the third to fourth layer of postmitotic neurons as seen in whole mounts (e.g. Fig. 1F) and histological sections (data not shown). This strongly suggests that PROS protein is also down regulated as the GCs move to deeper layers in the OPC while differentiating into neurons.

### Loss of *prospero* function increases mitotic activity and inhibits neurogenesis in the postembryonic brain

The transient upregulation of *pros* mRNA as well as the high levels of PROS protein in newly born neurons (GCs) suggested a possible novel role for PROS in the generation of postmitotic cells during postembryonic neurogenesis. To investigate this, we studied the effects *pros* loss-of-function (LoF) and gain-of-function (GoF) on proliferation and neurogenesis on postembryonic brain development.

**Figure 3:**
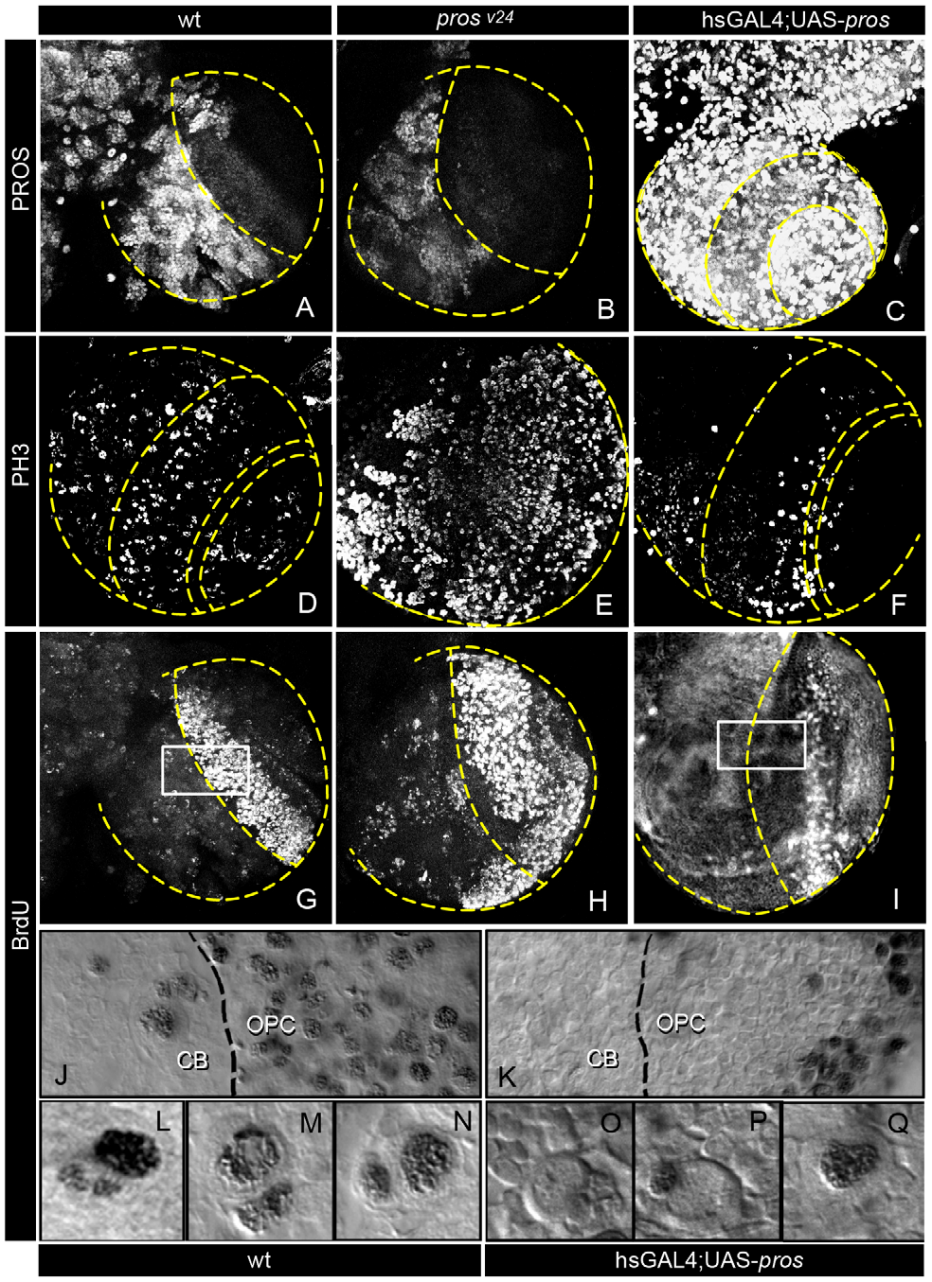
Alterations in neural proliferation caused by LOF and GOF of *pros*. A–I. Confocal projections taken through the OL of *w*t, *pros^v24^*, and *hsGal4;UAS-pros* late third instar larvae from a ventro-anterior orientation showing immunostaining for PROS, BrdU and PH3, as indicated. Notice the important increase in cell immunostained for both markers in *pros^v24^* and the clear decrease in *hsGal4;UAS-pros*. J,K. View of the CB/OPC border of the OL of wt and *hsGal4;UAS-pros*, respectively , showing an important decrease of DAB labeled BrdU immunostained cells in the mutant. L.Q. High magnification images of representative examples of BrdU immunostained CB NBs and its progeny. Most often NBs of *hsGal4;UAS-pros* lacked BrdU labeling (O,P) but GMCs frequently show it (P). Exceptionally, very few CB NBs of *hsGal4;UAS-pros* larvae exhibited BrdU labeling (Q).

Since *pros* null mutants are embryonic lethal, we first studied mitotic activity in *pros^v24^*, a strong hypomorphic allele [27] that reduces PROS expression in the larval OLs (Fig. 3A, B). Our findings revealed a marked increase in the number of mitotically active (proliferating) cells in the larval brain of *pros^v24^* hypomorphs compared to *wt* as determined by PH3 and BrdU immunolabelling (Fig. 3D, E G, H). Similar findings were obtained with Cyclin E immunolabeling (data not shown). Conversely, overexpression of PROS with the Gal4/UAS system [35] in *hs-Gal4;UAS-pros* larval brains resulted in a marked reduction in the number of mitotically active cells compared to *wt* when monitored with the same markers (Fig. 3D, F, G, I). This reduction in the number of mitotically active progenitor cells was seen both in the OPC and in the CB (Fig. 3J–Q). In the embryonic CNS, *pros* LoF also causes overproliferation but the supernumerary cells are eliminated by apoptosis [26]. In contrast, no increase in apoptosis was detected in the larval brain of *pros^v24^* hypomorphs as monitored by the expression of activated CASPASE-3 (Figure S1).

**Figure 4:**
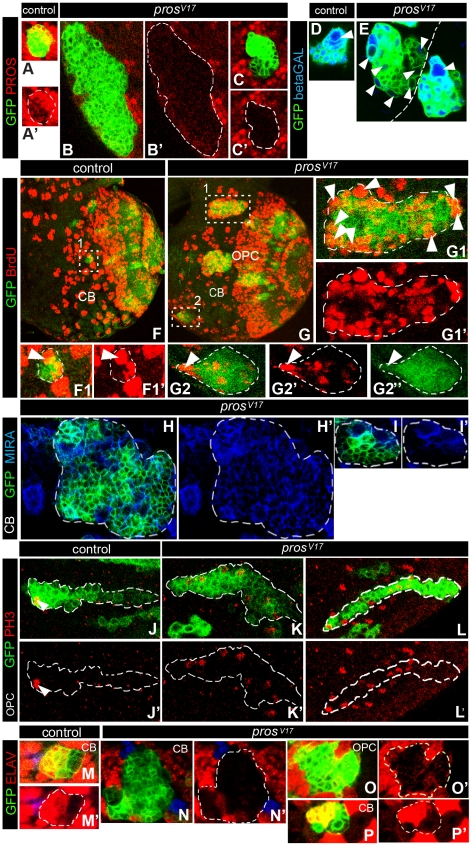
Clonal analysis of the LOF of *pros* unravels two differential phenotypes in the larval CNS. A,B,C. High magnification images of representative examples of *wt* and *pros^v17^* clones labeled with GFP and PROS. Note the larger size and the lack of PROS immunolabeling in the mutant clones compared to the *wt* clones. D,E, High magnification images of representative examples of *wt* and *pros^v17^* CB clones with double immunostaining for membrane GFP and nuclear βGalactosidase. E. Two neighbor *pros^v17^* clones. Note that the medium size mutant clone on the right side is larger than the *wt* clone (D) but also contains a single large putative NB (arrowhead). In contrast, the larger mutant clone on the left side in E contains multiple large cells. F, G. Representative examples of BrdU immunostained CB *w*t and *pros^v17^* clones. F1. The *wt* clone contains a labeled NB and one attached GMC, which is also labeled. Although the two mutant clones (G1,G2) contain multiple BrdU labeled nuclei, a difference between them can be appreciated: the large clone exhibits multiple large BrdU labeled nuclei distributed throughout the clone (G1) while the smaller *pros^v17^* clone harbors a single large nuclei located in one side (G2, arrowhead) and several small ones inside it. H,I. Two representative examples of the two types of *pros^v17^* clones immunostained with MIR. Notice that while in the large clone all the cells are MIRA+ (H), in the small one, only the large cells are MIRA+(I). J–L. High magnification images of representative examples of PH3 immunostained OPC *w*t and *pros^v17^* clones. Notice the single large labeled mitotic nuclei located on one side of the wt clone and the two different morphologies of the *pros^v17^* clones: the smaller one with elongated shape, similar to the wt clone (J) although with multiple PH3 labeled nuclei (L), and the large one with amorphous shape and multiple large PH3 labeled nuclei (K). M–P. Representative examples of *wt* and *pros^v17^* clones immunostained with GFP and ELAV. CB (N) and OPC (O) mutant clones lacked ELAV immunostaining. Nevertheless, very few small mutant clones showed strong ELAV+ positive cells (P) similar to wt clones (M).

To more precisely characterize the effect of *pros* LoF at the cellular level, we generated null-mutant MARCM NB clones in the developing postembryonic brain using *pros^v17^*. In accordance with previous reports [22]–[24], we found that most *pros^v17^* mutant clones in the larval brain were significantly larger than control *wt* clones (Fig. 4.A–C). This larger clone size was due to an increase in the number of mitotically active cells as judged by BrdU incorporation (Fig. 4F,G) and PH3 immunolabeling (Fig. 4J–L).

In the CB, two main types (A and B) of these *pros^v17^* mutant clones were recovered in an approximate 3A∶1B ratio (65 clones of 24 brain hemispheres). Clones of type A, although larger than *wt* clones, were similar to these in that they also contained one or two large cells located on one side of the cluster which seem to correspond to the NB and/or GMC (Fig. 4D, E-right clone). However, in addition to these large BrdU labeled cells, the mutant type A clones also contained several scattered small BrdU labeled nuclei (Fig. 4, F1, G2). Also similar to wt lineages, in type A *pros* clones the MIRA marker was strongly expressed in the large NB and the attached GMC but only weakly if at all in the small cells located at a distance from the NB (Fig. 4I). In contrast, type B clones were very large and mostly contained large-to-medium sized cells that were scattered throughout the clone (Fig. 4E-left clone). Moreover, all the cells of these type B clones showed strong MIRA labeling (Fig. 4H) and most of them were also BrdU-labeled (Fig. 4G1). Two comparable types of *pros^v17^* clones were also recovered in the OPC in an approximate 4A∶1B ratio (88 clones of 11 brain hemispheres). Thus, type A clones maintained the typical elongated shape of *wt* OPC clones, although in addition to the single mitotic NB detected in *wt* clones, they harbored several mitotic cells while type B clones were larger, relatively amorphous and contained multiple scattered large mitotic cells (Fig. 4J–L,O).

All type B clones and the majority of type A clones in the CB (16 clones, 5 brain hemispheres) abolished or showed a marked reduction in the expression of the pan-neuronal marker ELAV, indicating that the generation of neurons in these clones was inhibited (Fig. 4 M–O). This is in accordance with previous reports [22], [24]. Nevertheless, we occasionally (1/16 clones) found small *pros^v17^* clones that were similar in size to *wt* clones and in which the expression of ELAV appeared to be normal (Fig. 4P).

Taken together, these findings indicate that the LoF of *pros* results in an increase in proliferative mitotic activity and a decrease in the generation of neuronal cells in most postembryonic NB lineages of the CB and OPC although producing two different phenotypes possibly related to a dual role of PROS on cell cycle regulation and cell specification, as we discuss later.

### *prospero* regulates the expression of the cyclin kinase inhibitor *dacapo* in nascent larval CNS neurons

To determine if PROS plays a role in inhibiting cell cycle progression in new born neurons of the postembryonic brain, we took advantage of the fact that termination of mitotic activity in the larval OL is known to be controlled by the expression of the CKI *dacapo* (*dap*) [36]. We reasoned that if PROS could arrest proliferation in new born GCs, it might do so by regulating *dap* expression in these cells.

**Figure 5:**
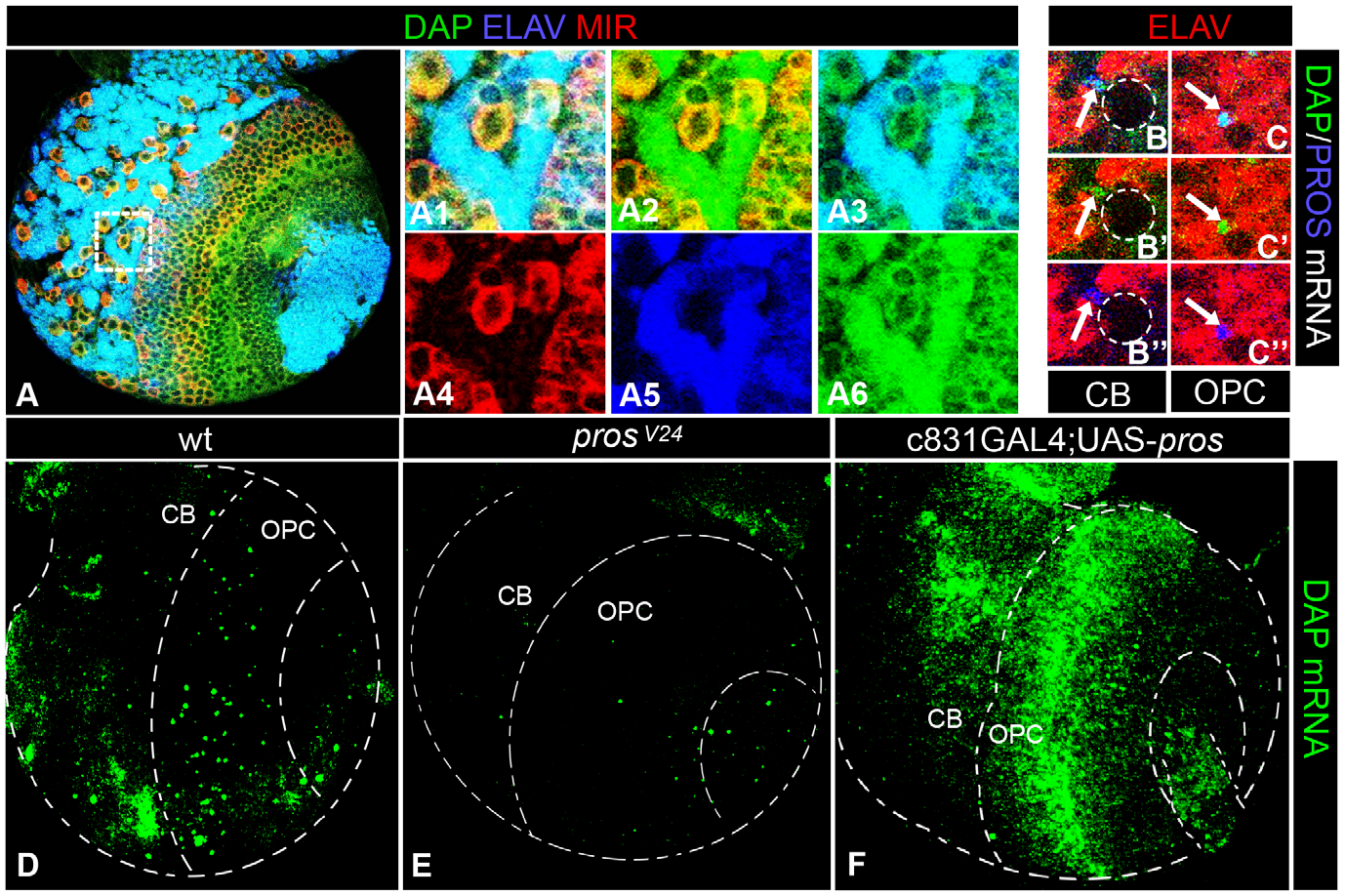
Changes in the expression of *dacapo* by LOF and GOF of *pros*. A. Confocal projection taken from a ventro-anterior orientation through the OL of a *w*t late third instar larvae showing immunostaining for DAP, MIR, and ELAV. A1–6. High magnification of the cell cluster framed in A showing individual and combined DAP/MIR/ELAV immunostainings. Notice that DAP signal is stronger in the nucleus of ELAV+ (GC) cells than in the cortex of MIR+ (NB and GMC) cells. B–D. Confocal projections of the OL of *w*t, *pros^v24^*, and *c831Gal4;UAS-pros* late third instar larvae showing DAP mRNA expression (FISH). Notice the presence of scattered labeled cells in the CB and OPC of wt OL, which are almost lacking in *pros^v24^* and, conversely, greatly increase in *c831Gal4;UAS-pros*. B1,2. Two representative examples of single cells coexpressing PROS and DAP mRNAs near a CB and an OPC NB, respectively. Notice that in both cases the NBs are deprived of both mRNAs.

To investigate this possibility, we first determined if *dap* is expressed in postmitotic neurons by immunocytochemical studies combined with cell-specific marker labeling. These experiments showed that DAP and PROS have similar expression patterns in cells of the postembryonic brain. Thus, DAP is much more strongly expressed in the nuclei of GCs as compared to NBs and GMCs where its expression is cytoplasmic and considerably weaker (Fig. 5A, A1–6). Moreover, in agreement with previous data [36], we found that *dap* mRNA was strongly expressed in scattered cells of the late third instar larval OL (data not shown). Since this expression pattern was reminiscent of that of PROS, we analyzed the possible co-expression of *pros* and *dap* by double FISH. These experiments showed that *pros* and *dap* mRNAs are indeed co-expressed in single GC-like cells in both the CB and OPC (Fig. 5B, C). This suggests that the expression of both genes is (transiently) upregulated in newborn GCs.

Next we carried out genetic LoF and GoF experiments to determine if PROS can regulate the expression of *dap* in these cells. Partial LoF in *pros^v24^* hypomorph mutants resulted in a marked decrease in *dap* mRNA expressing cells in the OL and CB (Fig. 5D, E). Conversely, PROS overexpression in *c831Gal4*; *UAS-pros* postembryonic brains resulted in a substantial increase in *dap* mRNA expressing cells in the OL (Fig. 5D, F).

Taken together, these findings indicate that PROS regulates the expression of *dap* in new born prospective neurons in the postembryonic brain. This supports the notion that PROS inhibits cell cycle progression in nascent GCs through DAP.

### *prospero* represses the expression of *deadpan* in the larval NB progeny

Since *dap* was not identified as a direct target of PROS in a genome-wide *in vivo* target gene identification analysis [21], it is unlikely that PROS can regulate the transcription of *dap* directly. Nevertheless, since PROS is required to terminate the embryonic expression of the pan-neural bHLH transcription factor *deadpan (dpn*) [26] which is a suppressor of *dap* expression in the larval OL [36], we hypothesized that DPN might mediate the upregulation of *dap* expression induced by PROS. To investigate this, we carried out LoF and GoF experiments to determine if PROS can regulate DPN expression in the postembryonic brain.

**Figure 6:**
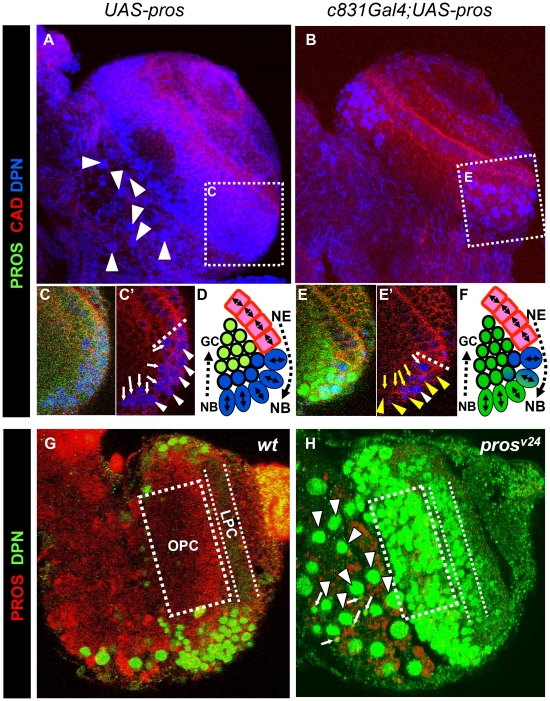
Changes in the expression of *deadpan* by LOF and GOF of *pros*. A,B. Confocal projections over 60 µm of the ventro-anterior side of the OL of *w*t and *c831Gal4;UAS-pros* late third instar larvae showing DPN and DE-CADHERIN (CAD) protein expression. Notice that many large DPN+ CB NBs (arrowheads) are missing in the *c831Gal4;UAS-pros* lobe which also shows an important decrease in DPN+ cells in the OPC. C,E. Single confocal images taken, as indicated in the framed areas of A and B, at a medial level of the most anterior part of the OPC. The dotted line marks approximately the border between the neuroepithelial (NE) cells and the NBs of the OPC as indicated by the sharp decrease in the expression of CAD and the increase of DPN. D, F. Schematic representations of the expression of CAD (red), DPN (blue), and PROS (green) observed in C and E, respectively. Notice that in the wt, the expression of DPN is very high in NBs (white arrowheads) and is maintained in their closest daughter cells (arrows) as they are asymmetrically generated inside the lobe. In contrast, in the *c831Gal4;UAS-pros* OL, although with a few exceptions (white arrow and arrowhead), DPN decays or almost disappears in most NBs (yellow arrowheads) and their closest daughter cells (second cell layer from the surface, yellow arrowheads) where PROS expression is driven at high level. These effects are in agreement with the c831-Gal4;UAS-GFP expression pattern (see Figure S2 for details). Notice that the intensity of PROS signal in the control sample OPC (C) is rather weak because the image acquisition intensity was set up at low level to avoid saturation in the PROS overexpressing sample (E). G, H. Single confocal images taken approximately at 20 µm from the ventro-anterior surface of the OL of *w*t and *pros^v24^* late third instar larvae showing expression of PROS and DPN. Notice the appearance of DPN expression in the cells located inside the OPC (framed area) and LPC in *pros^v24^*, which are both deprived of DPN+ cells in the *wt* OL. The *pros^v24^* CB also exhibits an increase in the presence of large DPN+ NBs located away from the lobe surface (arrowheads) and of small DPN+ daughter cells (small arrows) located nearby them.

In the *wt* larval brain, and as reported previously [1], [24], [37], we observed that DPN expression in the CB was mostly restricted to NBs (Fig. 6A, G) while in the OPC we found that DPN was highly expressed in the NBs located at the surface and, depending on the ventro-dorsal/anterior-posterior position, decays more or less rapidly in the daughter cell populations that move from the periphery towards the center of the OL as they are generated by the NBs (Fig. 6C′,D). We found that the ectopic expression of PROS in NBs and their early progeny using the *c831Gal4* driver (see Figure S2 for details of expression pattern) strongly reduced the expression of DPN in cells of the CB and OPC (Fig. 6A, B). Close inspection of the OPC in these experiments revealed that DPN expression was greatly reduced or abolished in most NBs and their progeny where PROS expression was induced at high level (Fig. 6E,E′,F). Conversely, in *pros^v24^* mutants we observed the ectopic expression of DPN in the progeny of some CB NBs and in most of the progeny of NBs in the OPC (Fig. 6G, H).

These results indicate that PROS represses the expression of DPN in the NBs progeny during postembryonic brain development. Since DPN is known to repress *dap* expression (at least in the larval OL) [36], these findings support the hypothesis that DPN mediates the upregulation of *dap* expression induced by PROS.

## Discussion

### A transient upregulation of *prospero* promotes the cell cycle exit of *Drosophila* postembryonic CNS neurons

During development, cell cycle progression must be coordinated with the regulation of cell specification and differentiation. The underlying mechanisms of coordination are likely to be particularly complex during neural development due to the enormous cell diversity in the brain. In *Drosophila*, these mechanisms have been well studied during embryonic CNS development. In embryonic neurogenesis, the homeodomain transcription factor PROS is expressed in the NB but it does not enter the nucleus due to its binding to the carrier protein MIRA, which localizes to the cell cortex. This interaction facilitates the segregation of PROS from the parent NB to the GMC during asymmetric NB division. In the GMC, PROS is released from its carrier and translocates to the nucleus where it plays a binary role as a cell fate determinant [38]–[41], and as a promoter of terminal differentiation [20], [21], [42].

**Figure 7:**
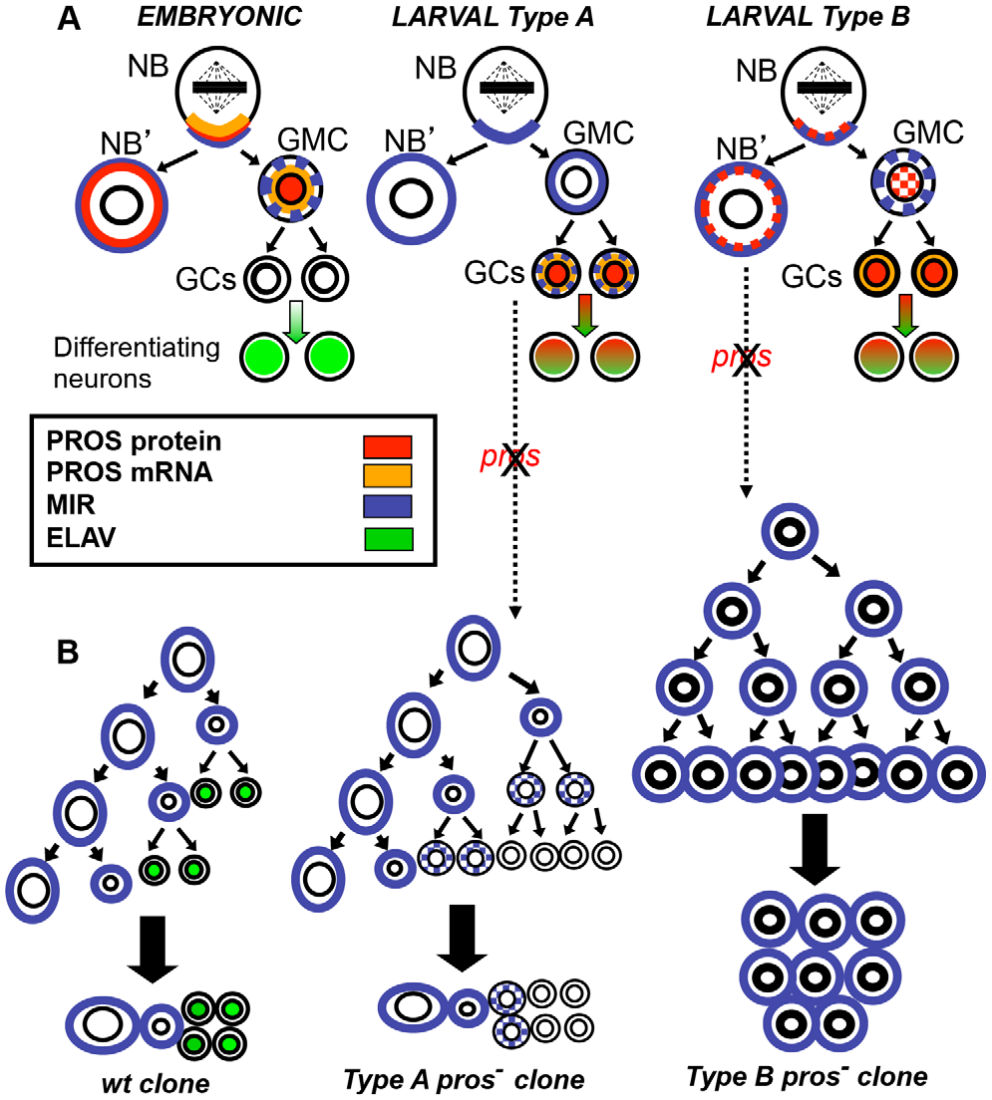
Summary of cellular expression pattern of PROS in the larval CNS and lineage alterations in *pros* mutant clones. A. Schematic representation of the expression patterns of PROS in embryonic and larval CNS NB lineages. Both embryonic and larval type I NBs divide asymmetrically to self-renew and produce a GMC that undergoes a terminal division to generate two postmitotic GCs. In embryonic lineages, *pros* mRNA and PROS protein are asymmetrically localized in the NB during mitosis, segregated to the GMC during NB division, and down regulated in GCs. Apart from the dorso-medial and mushroom bodies NBs, which do not express PROS, we have observed two different PROS expression patterns in the CB and OPC. In type A, PROS protein and mRNA were only detected in new born GCs. In contrast, in type B, we observed expression and asymmetric segregation of PROS in NBs at low level and at high level in newborn GCs. B. Schematic representation of the cellular and molecular phenotypes found in *pros* MARCM clones. In *wt* clones, after three rounds of self-renewing divisions, the clone would consists on a NB, a GMC and four ELAV+ GCs. The LoF of *pros* in lineages with type A PROS pattern will preclude cell cycle exit and differentiation of GCs. Thus, after three rounds of divisions, the resulting (type A) clone will consist on a MIR+ NB, a MIR+ GMC, and six small ELAV- cells with decreasing level of MIR depending on the number of divisions occurred. In lineages with type B pattern, the LoF of *pros* will preclude the specification of the GMCs and will result after three rounds of divisions in a (type B) clone consisting on multiple large NB like MIR+/ELAV- cells.

It has been reported that PROS is similarly expressed and asymmetrically segregated during the proliferative activity of (type I) NBs in the larval CB [1], [23], [24], [43] although it does not seem to be expressed in CB dorso-medial lineages (type II) [34] NBs. However, as we show here, during postembryonic neurogenesis, in the majority of larval CB and OPC neuronal lineages, *pros* expression is transiently upregulated in new born prospective neurons (GCs), in addition to its earlier expression and asymmetric segregation in some larval NBs. This is clearly different from the situation in embryonic lineages where *pros* is only transcribed in NBs [44], [45], and PROS protein is downregulated in GCs after the division of their parent GMC [26]. A summary scheme of these different situations in embryonic versus postembryonic neurogenesis is shown in Fig. 7A.

This transient expression in most newborn postembryonic neurons shortly after the division of the GMC implies a novel role of PROS in postmitotic cells. We postulate that this role is to inhibit cell cycle progression and promote cell cycle exit. Our PROS GoF and LoF experiments support this notion. PROS GoF induces proliferation arrest and PROS LoF results in supernumerary cells with sustained expression of cell cycle markers, indicating an inability to withdraw from the cell cycle.

### Differential roles of *prospero* in GMCs and GCs during postembryonic neurogenesis in *Drosophila*

In addition to the marked difference in PROS expression in postmitotic GCs during embryogenesis versus postembryonic neurogenesis (PROS is undetectable in embryonic GCs and high in postembryonic GCs), there are other functional differences in PROS action during embryonic versus postembryonic CNS development. For example, in *pros* mutant embryos, overproliferation is followed by abundant apoptotic cell death among the supernumerary cells [20]. By contrast, we find no increased cell death in the larval OL of *pros* mutants. Moreover, while PROS and DAP seem to act in parallel to end the cell cycle in the embryonic CNS [20], DAP appears to act downstream of PROS in larval CNS neurons, as shown here (see discussion below). These initial findings suggest that further differences between the functions of PROS during embryonic and postembryonic CNS neurogenesis may exist and should be considered.

The fact that PROS protein is present in embryonic GMCs (intermediate progenitors) but not in embryonic GCs (prospective neurons) [25], [26], suggests that in the embryonic CNS, PROS initiates the end of mitotic activity in the GMC rather than in the GC. Accordingly, it has been proposed that the GMC is a transition state between the proliferating NB and the differentiating neuron that provides a window in which PROS represses stem cell-specific genes and activates differentiation genes [21]. Nevertheless, it is not well understood how the GMC can go through its terminal cell cycle in spite of the repressive action of PROS on cell cycle regulators.

Our results strongly suggest that in postembryonic neurogenesis PROS acts not only in the GMC progenitor but also in the postmitotic GCs produced by the GMC. Thus, our analysis indicates that there are two main *pros* expression pattern subclasses among CB type I and OPC NB lineages (Fig. 7A). For the shake of simplicity we have called them A and B. In type A, PROS is expressed in GCs after the division of GMCs while in type B, PROS is first expressed at low level in the NB and asymmetrically segregated to the GMC, and afterwards, upregulated in new born GCs. We interpret that these two subsets of expression patterns correlate well with the two main phenotypes found in *pros* mutant clones (Fig. 7A, B). Thus, the LOF of *pros* in NBs with type A PROS expression appears to preclude cell cycle exit of GCs which, consequently, continue dividing and do not differentiate, yielding a type A clone composed of a single NB, a GMC and several small mitotic cells. By contrast, in lineages with type B PROS expression, the LOF of *pros* seems to cause primarily a change in the fate of the putative GMC that behaves like a NB maintaining the expression of asymmetric division genes (such as MIRA) and overproliferating, to yield a type B clone composed of multiple large NB like cells (Fig. 7A,B).

Hence, we postulate that during postembryonic neurogenesis PROS functions in two sequential phases in type I NB lineages, first as cell fate determinant in some GMCs and later as cell cycle repressor in most GCs. Furthermore, we favor the idea that the different roles of PROS in postembryonic GMCs versus postembryonic GCs might be related to the higher level of expression observed in GCs compared to GMCs. Thus, high levels of PROS might be required to definitively withdraw the GCs from the cell cycle, while low levels might be sufficient to specify GMCs and modulate their cell cycle. The higher level of DAP protein in postembryonic GCs in relation to their parent GMCs and NBs is consistent with this hypothesis. The strong burst of PROS at the end of NB proliferation in ventral ganglia of early pupae [46] is also in agreement with the idea that high levels of PROS are required to stop proliferation. Furthermore, it has been recently shown that the missexpression of PROS at high level suppresses proliferation in type II larval brain NBs lineages without apparent change in their identity [47].

Taken together, all of these findings imply that different developmental strategies have been selected to couple cell fate decisions and cell cycle regulation during embryonic and postembryonic neurogenesis through the same effector, PROS. It is possible that this change in strategy is a consequence of the evolutionary adaptation to regulate the production of a large number of equivalent neurons in postembryonic lineages in contrast to embryonic neurogenesis where a much more limited set of specific neurons are generated in each lineage through GMC divisions.

### *deadpan* and *dacapo* act in sequence downstream of *prospero* to regulate the cell cycle exit of *Drosophila* post-embryonic neurons

**Figure 8:**
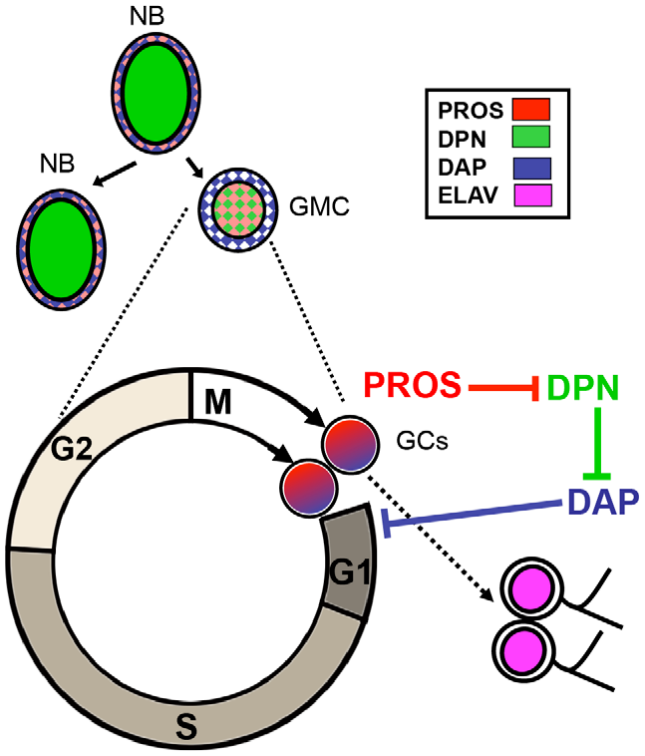
Regulation cascade downstream of PROS in the cell cycle exit of postembryonic neurons. Schematic model for the transcriptional regulation cascade downstream of PROS in the control of cell cycle exit of postembryonic neurons. After the division of the GMC, *pros* expression is upregulated in the new born GCs. This induces the full down regulation of *dpn* and, consequently derepresses the expression of *dap*, which induces the cell cycle exit of the GCs that begin to differentiate expressing the neuronal marker ELAV.

We have here shown that PROS is coexpressed with DAP in new born prospective neurons and, moreover, we have found that *pros* is sufficient and it is required for the expression of *dap* in these larval brain neuronal precursors. The *dap* gene encodes a member of the Cip/Kip family of CKIs with homology to mammalian *p27^kip1^*. This family of CKIs has been implicated in mediating cell cycle exit prior to terminal differentiation. They function by binding and inhibiting G1/S cyclin dependent kinase complexes (reviewed by [48]). There is compelling data supporting a role of DAP in cell cycle exit during *Drosophila* embryogenesis [31], [49]. In *Drosophila* embryonic NB lineages, *dap* expression becomes apparent just before the terminal neurogenic division of the GMC [31]. In contrast, we have here shown that *dap* is upregulated in new born postembryonic neurons. Consistent with a role in the termination of cell proliferation, *dap* expression in the larval OL has been tightly correlated with cells ending proliferation [36]. Interestingly, PROS is required to terminate cell proliferation during embryonic neurogenesis [20] and it has been shown to be involved in the regulation of *dap* expression in the embryonic nervous system [42]. Thus, our results provide support to the idea that PROS promotes the cell cycle exit of post-embryonic GCs by upregulating the expression of *dap*. Our data also suggest that this upregulation of *dap* is mediated by inhibiting the expression of DPN (see Fig. 8 for a schematic summary). DPN is an essential panneural bHLH transcription factor, which has been previously shown to be a suppressor of *dap* expression in the larval OL [36]. Indeed, the *dpn* gene contains consensus PROS binding sites [21] and PROS has been shown to be required to terminate the expression of *dpn* in the embryo [26].

Abundant data from diverse experimental systems have also implicated *Prox1*, the vertebrate orthologue of *pros* [50], in cell cycle exit regulation. Thus, studies in mice have shown that *Prox1* appears to be required for the temporal expression of *p27kip1* in lens fiber development [51]. Similarly, *Prox1* regulates cell cycle exit in the embryonic mouse retina preceding the upregulation of p27^Kip1^ [52]. Furthermore, *Prox1* is expressed in early differentiating mouse CNS neurons [53]. Together, these data stimulate to study whether a similar sequential cascade of genes downstream of *Prox1* regulates the cell cycle exit of vertebrate CNS neurons.

## Supporting Information

Figure S1 **The LoF of** ***pros*** **does not induce cell death in the larval CNS.** A,B. Confocal projections taken from a ventro-anterior orientation through *wt* and *pros^v24^* late third instar larvae OL showing immunostaining for activated CASPASE 3 (CASP3). Notice that there is no apparent change in CASP3 immunolabeling.
(TIF)

Figure S2 **Expression pattern of c831-Gal4;UAS-GFP in late larval brain.** A,B. Confocal images of a c831Gal4;UAS-GFP third instar larvae brain hemisphere taken from a ventro-anterior point of view at two different levels: close to the surface (A) and aprox. 12 µm inside the lobe (B) showing immunostaining for GFP, MIR, and ELAV. A1. High magnification view of the CB cell cluster framed in A showing expression of GFP in a MIR+/ELAV- NB (arrowhead) and its attached GMC (empty arrowhead), as well as in the MIR-/ELAV+ surrounding GCs. A2. High magnification of cell cluster framed in A at the surface of the OPC showing low expression of GFP in the MIR+ OPC NBs (arrowheads) and high expression in the progeny that is downregulating MIR expression but do not express ELAV yet (GMCs and new born GCs; empty arrowheads). B3. High magnification of the cell cluster framed in B inside of the OPC showing high expression of GFP in ELAV+/MIR- cells (differentiating GCs). C. Confocal image of a c831Gal4;UAS-GFP third instar larvae OL taken at a medial level (equivalent to those of Fig. 6C,E) showing expression of GFP, DE-CADHERIN (CAD), and PATJ. D. High magnification view of the framed area in C around the most anterior part of the OPC. E. Schematic representations of the cell types and expression patterns found in D. The expression of GFP begins in NBs as they delaminate and increases as NBs move tangentially from the neuroepithelium (NE), which is identified by the high expression of CAD and PATJ. The expression of GFP is further increased in the NB daughter cells as they move inside the OL.
(TIF)
