# Supplementary figures and images for: A Transient Expression of Prospero Promotes Cell Cycle Exit of Drosophila Postembryonic Neurons through the Regulation of Dacapo
Source: PLoS One. 2011 Apr 28;6(4):e19342. doi: 10.1371/journal.pone.0019342 (PMC3084296; doi:10.1371/journal.pone.0019342)

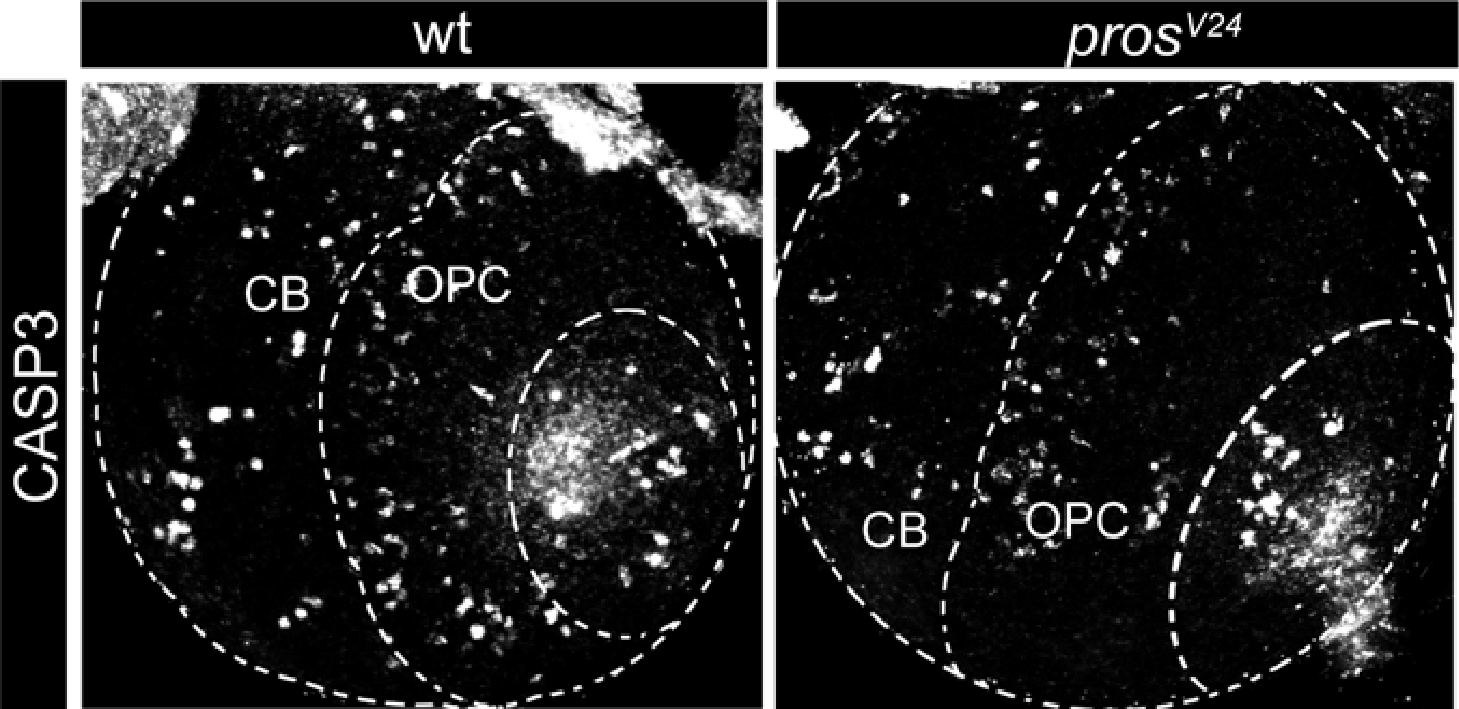

Supplement: Figure S1 — The LoF of pros does not induce cell death in the larval CNS. A,B. Confocal projections taken from a ventro-anterior orientation through wt and prosv24 late third instar larvae OL showing immunostaining for activated CASPASE 3 (CASP3). Notice that there is no apparent change in CASP3 immunolabeling. (TIF) [file pone.0019342.s001.tif]

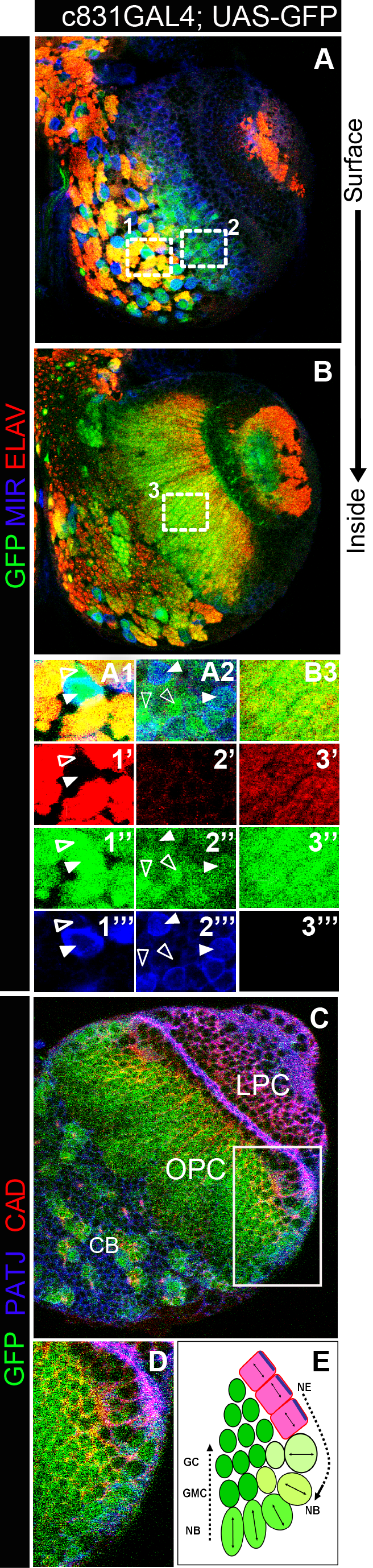

Supplement: Figure S2 — Expression pattern of c831-Gal4;UAS-GFP in late larval brain. A,B. Confocal images of a c831Gal4;UAS-GFP third instar larvae brain hemisphere taken from a ventro-anterior point of view at two different levels: close to the surface (A) and aprox. 12 µm inside the lobe (B) showing immunostaining for GFP, MIR, and ELAV. A1. High magnification view of the CB cell cluster framed in A showing expression of GFP in a MIR+/ELAV- NB (arrowhead) and its attached GMC (empty arrowhead), as well as in the MIR-/ELAV+ surrounding GCs. A2. High magnification of cell cluster framed in A at the surface of the OPC showing low expression of GFP in the MIR+ OPC NBs (arrowheads) and high expression in the progeny that is downregulating MIR expression but do not express ELAV yet (GMCs and new born GCs; empty arrowheads). B3. High magnification of the cell cluster framed in B inside of the OPC showing high expression of GFP in ELAV+/MIR- cells (differentiating GCs). C. Confocal image of a c831Gal4;UAS-GFP third instar larvae OL taken at a medial level (equivalent to those of Fig. 6C,E) showing expression of GFP, DE-CADHERIN (CAD), and PATJ. D. High magnification view of the framed area in C around the most anterior part of the OPC. E. Schematic representations of the cell types and expression patterns found in D. The expression of GFP begins in NBs as they delaminate and increases as NBs move tangentially from the neuroepithelium (NE), which is identified by the high expression of CAD and PATJ. The expression of GFP is further increased in the NB daughter cells as they move inside the OL. (TIF) [file pone.0019342.s002.tif]
